# Hollow fiber-based strain sensors with desirable modulus and sensitivity at effective deformation for dexterous electroelastomer cylindrical actuator

**DOI:** 10.1038/s41378-025-00878-7

**Published:** 2025-02-27

**Authors:** Yang Zhang, Keqi Deng, Tingting Shen, Yong Huang, Zhenjin Xu, Jinhui Zhang, Hang Jin, Xin Liu, Lida Xu, Lianjie Lu, Shiying Li, Daoheng Sun, Dezhi Wu

**Affiliations:** 1 Pen-Tung Sah Institute of Micro-Nano Science and Technology, Xiamen University, 361005 Xiamen, China; 2 Department of Mechanical & Electrical Engineering, Xiamen University, 361005 Xiamen, China; 3 Department of Ophthalmology, The First Affiliated Hospital of Xiamen University, School of Medicine, 361005 Xiamen, Fujian Province China

**Keywords:** Sensors, Structural properties

## Abstract

The electroelastomer cylindrical actuators, a typical representation of soft actuators, have recently aroused increasing interest owing to their advantages in flexibility, deformability, and spatial utilization rate. Proprioception is crucial for controlling and monitoring the shape and position of these actuators. However, most existing flexible sensors have a modulus mismatch with the actuation unit, hindering the free movement of these actuators. Herein, a low-modulus strain sensor based on laser-induced cellular graphitic flakes (CGF) onto the surface of hollow TPU fibers (HTF) is present. Through the electrostatic self-assembly technology, the flexible sensor features a unique hybrid sensing unit including soft HTF as substrate and rigid CGF as conductive path. As a result, the sensor simultaneously possesses desirable modulus (~0.155 MPa), a gauge factor of 220.3 (25% < ε < 50%), fast response/recovery behaviors (31/62 ms), and a low detection limit (0.1% strain). Integrating the sensor onto the electroelastomer cylindrical actuators enables precise measurement of deformation modes, directions, and quantity. As proof-of-concept demonstrations, a prototype soft robot with high-precision perception is successfully designed, achieving real-time detection of its deformations during the crawling process. Thus, the proposed scheme sheds new light on the development of intelligent soft robots.

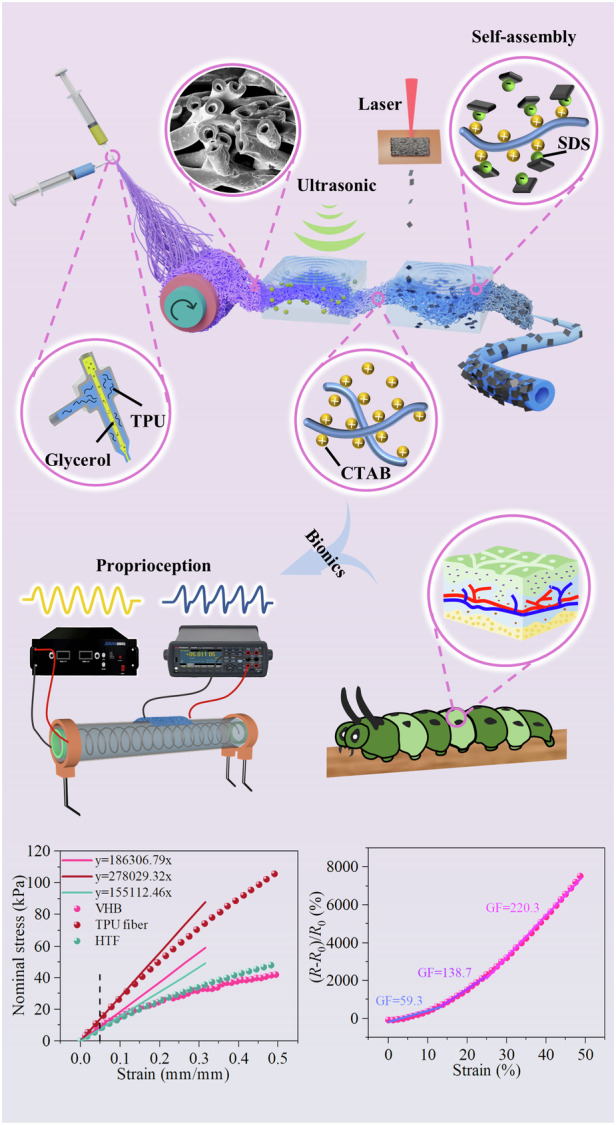

## Introduction

Soft actuator, an emerging field, has been extensively studied due to its motion flexibility, structural compliance, and human interaction safety^1,2^. Among various soft actuators, dielectric elastomer actuator (DEA) is regarded as the next-generation artificial muscle due to its fast response time (~ms), large deformation (>380%), and high energy density (3.4 MJ/m^3^)^3–5^. For better integration with practical demands, the cylindrical DEA configuration is introduced to follow a linear motion along the axial direction^6,7^. And it plays a pivotal role in diverse applications in fields such as soft robots, soft grippers, etc.^8^.

In general, a pair of electrodes of DEA can only achieve one actuation deformation mode^9^. To balance the dexterity and output performance of the actuator, three pairs of electrodes are arranged equally around the cylindrical axis^10^. In this case, the effective actuation strain of the actuator in this configuration is <50%. Despite the perfect adaptability and flexibility of cylindrical actuators, the viscoelastic properties of soft actuation units pose significant challenges in controlling and monitoring their deformation and position during motion, thereby compromising the effectiveness of the actuators^11,12^. To address this issue, it is necessary to integrate flexible strain sensors into soft robots for sensory feedback^13,14^.

Currently, there are two main types of flexible strain-sensing technologies for soft robots. A more common approach is to utilize the inherent characteristics of materials to achieve self-sensing functionality^15,16^. Increased voltage or extended operating time can elevate the leakage current in DEs, causing capacitance degradation and a reduced signal-to-interference-plus-noise ratio (SINR), which affects sensing accuracy^17^. Furthermore, the inherent viscoelasticity of VHB leads to poor consistency in the self-sensing signals of DEs^18^. To address these challenges, some researchers have achieved accurate detection of DEAs through algorithms, but this approach is only suitable for simple configurations of DEAs, such as conical configurations^19–21^. For the cylindrical DEAs prepared by coiling and stacking, the complexity of the model makes it difficult to implement an algorithmic compensation mechanism for accurately self-sensing deformation.

Another approach is to integrate functional fillers into inherently stretchable elastomers to form flexible sensors, which can effectively meet the sensing requirements of highly compliant soft robots^22,23^. Functional filles such as hydrogels, metals, carbon-based materials, and conductive polymers have been successfully combined with various elastomeric substrates, including polyethylene terephthalate, polydimethylsiloxane (PDMS), and silicone rubber^24–27^. Although these flexible sensors have a lower modulus, their mechanical properties are still slightly higher than those of the actuation unit, limiting the free deformation of soft robots^28,29^. In addition, these sensors still do not meet the application requirements of strain sensors for high sensitivity at low modulus^30^. Therefore, the top priority is to explore a strain-sensing technology that is coordinated with the mechanical properties of the actuator materials and exhibits high sensitivity within a small strain to detect the actuation behavior in real-time for better manipulation of objects.

Herein, a paradigm for low-modulus strain sensors based on laser-induced cellular graphitic flakes (CGF) on the surface of hollow TPU fibers (HTF) is presented. The flexible sensor features a unique hybrid sensing unit including soft HTF as substrate and rigid CGF as conductive path. For a more concise description, the strain sensor is represented next to the HTF–CGF sensor. This strategy demonstrates three major advantages: first, the hollow fiber configuration further reduces the modulus of the flexible sensing substrate; second, the method of anchoring CGF on the surface of HTF via an electrostatic self-assembly process allows the sensing unit to have better durability; third, the hybrid sensing unit composed of rigid CGF and soft HTF leads to the reduction of effective contact or even the formation of gaps between the graphite sheets under small strains, thereby disrupting the conductive network. The strain sensor designed using this strategy demonstrates a combination of desirable modulus (~0.155 MPa), a maximum gauge factor of 220.3 (25% < *ε* < 50%), fast response/recovery behaviors (31/62 ms), and a low detection limit (0.1% strain). The sensor was attached to an electroactive polymer acrylic film for electrical stimulation testing. The results indicate that the difference in actuation area between the actuator with/without the sensor is 12%. Subsequently, the sensor was integrated into the electroelastomer cylindrical actuator to accurately measure the deformation modes, directions, and quantity. Finally, a soft robot capable of real-time monitoring of its proprioceptive deformation has validated the feasibility of the approach. It is believed that this proposed strategy broadens the way for advanced flexible sensing technologies for soft robots.

## Results and discussion

### HTF–CGF sensor’s basic characterization

**Fig. 1:**
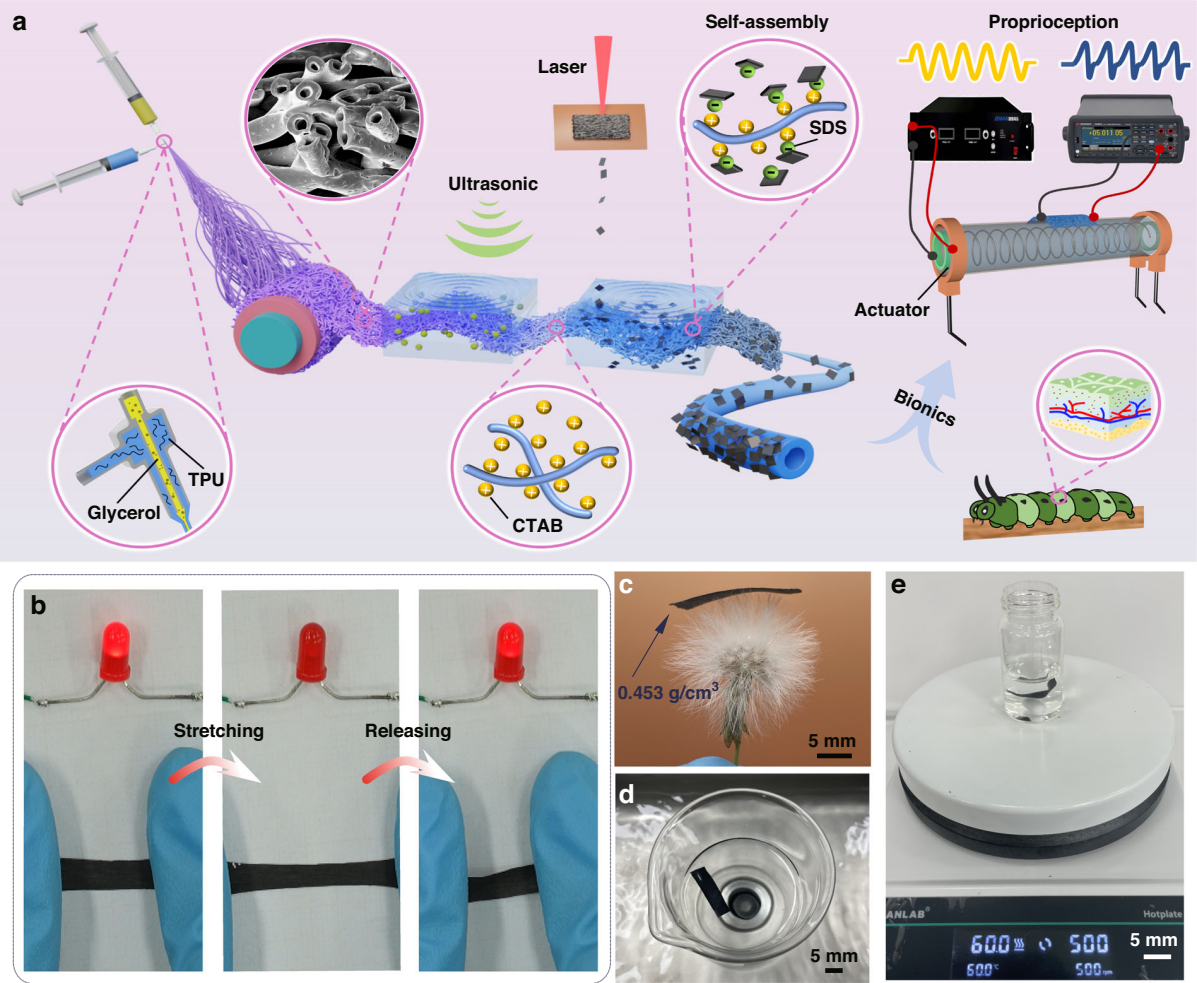
HTF–CGF strain sensor’s preparation technology and its based tests. **a** The sensor’s manufacturing strategy. **b** The luminance changes of the LED between stretching and releasing the HTF sensing unit. **c** Photograph of HTF–CGF on the crown of dandelion to emphasize its density. **d** Ultrasonic washing in deionized water, and **e** magnetic stirring in sodium chloride aqueous solution to highlight its durability

Figure 1a provides a schematic illustration of the HTF–CGF strain sensor preparation procedure, which is described in detail in the “Material and methods” section. HTF–CGF sensing unit prepared based on this strategy presents excellent electrical conductivity, as seen clearly in Fig. 1b. When the HTF–CGF sensing unit was connected with a light-emitting diode (LED), a significant change in brightness of the LED was observed under small tensile strain. Upon releasing the strain, the brightness of the LED increased again. This phenomenon demonstrates the enormous potential of the HTF–CGF sensing unit as a strain sensor. The hollow fiber is formed after the evaporation of glycerin remaining inside the fibers. This particular configuration endows the fiber with excellent lightweight properties, with a density of ~0.453 g/cm^3^. As illustrated in Fig. 1c, the HTF–CGF sensing unit was placed on the dandelions without bending the crown, showing its lightness.

To verify the excellent durability of the HTF–CGF sensing unit, it was subjected to rigorous treatments (Fig. 1d, e). Despite these harsh conditions, there were no visible black particles in those solutions, and no apparent damage was observed on the surface of the film. These results demonstrate that the HTF–CGF sensing unit has excellent electromechanical performance for practical applications.

**Fig. 2:**
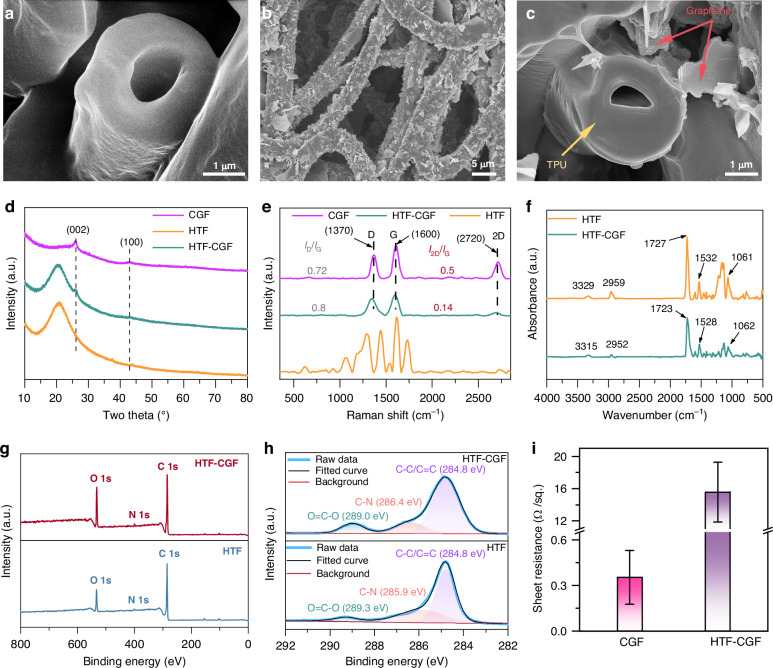
Characterization of HTF–CGF sensing unit. **a** Cross-sectional SEM image of the HTF sample. HTF–CGF sample’s **b** SEM image and **c** cross-sectional SEM image. **d** XRD and **e** Raman spectra of CGF, HTF, and HTF–CGF samples. **f** FTIR spectra, **g** XPS spectra, **h** XPS C1*s* spectra and **i** sheet resistances of HTF and HTF–CGF samples

A co-axial electrospinning technology is used to fabricate hollow fibers to accommodate flexible substrates with low modulus. Glycerol possesses a high viscosity and excellent volatility, making it an ideal core solution with slow diffusion rates during co-axial electrospinning. The TPU solution is selected as the shell solution. Then, the coaxial jet of two different solutions can realize stable electrospinning. HTF was successfully prepared by immersing the composite fiber membrane obtained by coaxial electrospinning into deionized water and stirring to remove the excess glycerol. As seen clearly in Fig. 2a, a hollow structure exists inside the fiber. And the outer diameter of a single fiber is ~2.3 μm.

To enhance the electrical characteristics of the HTF, the conductive fillers were attached to fibers. The common methods for graphene preparation mainly include epitaxial growth^31^, chemical vapor deposition (CVD)^32^, stripping^33^, and laser-induced graphene (LIG)^34^. Among them, the LIG method has been widely reported due to the advantages of low cost and simple preparation process. Extensive research has presented that porous graphene structures are available through LIG technology, facilitating the production of graphene-based electronics^35–37^. Figure S1 shows porous CGF obtained via the LIG technique.

Then, CGF was modified with sodium dodecyl sulfate (SDS) to impart a negatively charged, enabling it to self-assemble onto the surface of HTF modified with cetyltrimethylammonium bromide (CTAB) to impart a positive charge, thereby preparing HTF–CGF films. Subsequently, the conjecture is confirmed by zeta potential (Fig. S2). The zeta potentials of SDS/CGF and CTAB solutions are −44.1 and 47.3 mV, respectively, which contribute to the electrostatic self-assembly process of HTF–CGF films. SEM observations reveal that CGF successfully wraps the surface of each fiber, constituting a satisfactory interface between CGF and HTF (top and cross-sectional views in Fig. 2b, c).

The molecular and morphological characteristics and the transformation mechanism of the CGF, HTF, and HTF–CGF samples were investigated. The XRD patterns of these samples are given in Fig. 2d. CGF sample exhibited peaks at 25.9° and 43° due to (002) and (100) reflections originating from an in-plane structure^38,39^. In contrast to the peaks of the HTF sample, the HTF–CGF sample shows the same peak as the CGF sample, which indicates that CGF was wrapped around the surface of the HTF. To further support this finding, the Raman spectra were chosen to analyze the structural characteristics of these samples. As illustrated in Fig. 2e, CGF and HTF-CGF samples exhibited D bands at 1370 cm^−1^, G bands at 1600 cm^−1^, and 2D bands at 2720 cm^−1^, implying that CGF is attached to HTF–CGF^40,41^. These graphene structures appear to be multilayered and disordered fringes based on the *I*_2D_/*I*_G_ ratio of 0.14 and *I*_D_/*I*_G_ ratio of 0.8 for HTF–CGF samples (the *I*_2D_/*I*_G_ and *I*_D_/*I*_G_ ratios of CGF were 0.5 and 0.72, respectively).

XPS and FTIR spectra were adopted to further support the successful introduction of CGF. As shown in Fig. 2f, the characteristic peaks of HTF at 3329, 2925 cm^−1^ correspond to the stretching and bending vibrations of N–H and –CH, at 1730, 1532 cm^−1^ to the vibrations of –H–N–COO–, and at 1070 cm^−1^ to C–O–C bond^42–44^. In contrast to HTF, HTF–CGF samples exhibited slight shifts, suggesting that the amino and carbonyl groups of the HTF may be bonded to the functional groups of graphene through interfacial interactions to produce effective load transfer during dynamic stretching. Previous research has reported similar results^45^. In addition, the absence of new peaks for the HTF–CGF sample confirms that the addition of graphene retains the corresponding chemical structure of HTF.

The elemental composition of HTF and HTF–CGF samples were characterized by XPS. As seen in Fig. 2g, both HTF and HTF–CGF samples present three featured peaks at 532 eV (O1*s*), 399 eV (N1*s*), 285 eV (C1*s*), respectively. In the high-resolution C1*s* spectrum of the HTF–CGF sample (top panel in Fig. 2h), three deconvolved peaks are observed, corresponding to O=C–O (289.0 eV), C–N (286.4 eV), C–C/C=C (284.8 eV), respectively. For reference, the C1*s* spectrum of the HTF sample (bottom panel in Fig. 2h) consists of O=C–O (289.3 eV), C–N (285.9 eV), C–C/C=C (284.8 eV) species^46,47^. These indicate that after CGF is electrostatically self-assembled onto the surface of HTF, both the O=C–O and C–N bonds are shifted, which can be accounted for their interactions.

Indeed, in contrast to the non-conducting property of HTF, HTF–CGF possesses electrical conductively after the successful introduction of CGF. The average sheet resistance value obtained was 15.8 Ω/sq by the four-point probe method (Fig. 2i).

### Electromechanical performance and sensing mechanism of the sensor

One of the crucial indicators of the stretchable strain sensors is the sensitivity, which can be stated in terms of the gauge factor (GF). Its calculation formula is as follows:

1 $${\rm{GF}}=\frac{(R-{R}_{0})/{R}_{0}}{\varepsilon }$$

where *R* and *R*_0_ represent the tensile and initial resistances, respectively, *ε* is the tensile strain.

**Fig. 3:**
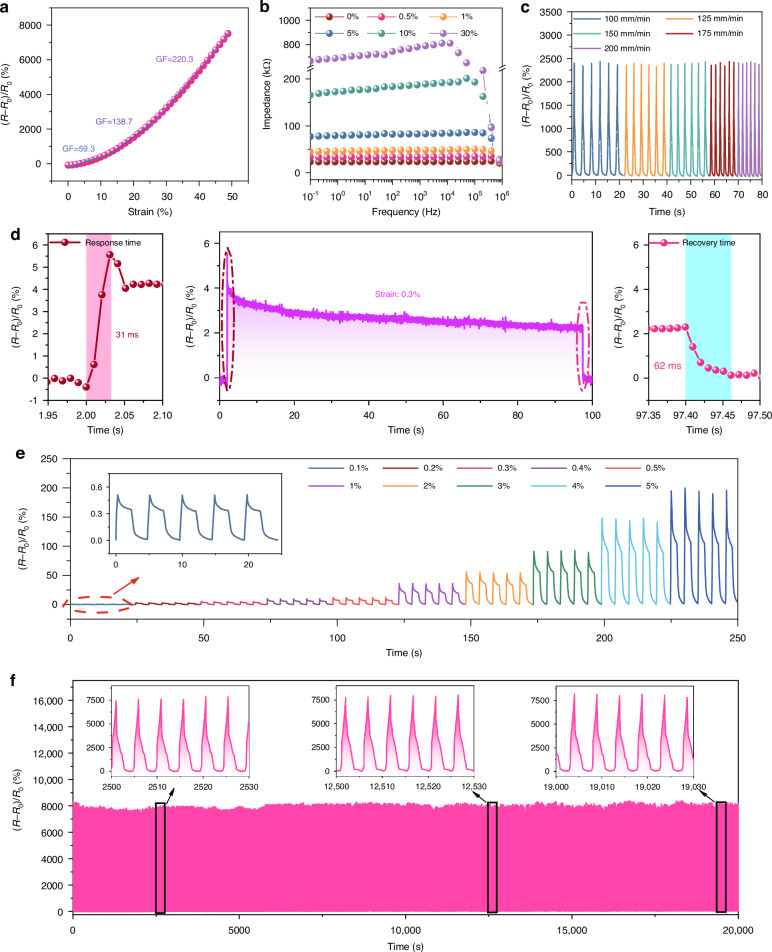
Basic sensing behaviors of the sensor. **a** Relative resistance changes versus strain. **b** The electrical impedances of HTF–CGF samples in the 0–30% strain range. **c** Relative resistance changes under various strain rates. **d** Response time under 0.3% strain loading/unloading. **e** Dynamic responses under different strains. Inset: responses under a slight strain of 0.1%. **f** Cyclic repeatability test

Figure 3a shows the variation of relative resistance versus applied tensile strain. HTF–CGF strain sensor reaches a GF value of 59.3 in the 0–15% strain range, 138.7 in the 15–25% strain range, and 220.3 in the 25–50% strain range. The high-frequency impedances of the HTF–CGF strain sensor under the initial and tensile states were investigated and shown in Fig. 3b. The stretching ratio is positively correlated with the impedance. The impedance at different stretch ratios tends to stabilize in the range from 0.1 to 10 kHz, and decreases slightly when the frequency exceeds 10 kHz. This demonstrates the sensor’s capability to adapt to dynamic loads. Figure 3c demonstrates the relative resistance change for different tensile velocities in the 0–25% strain range of the sensor. When the strain rate is below 200 mm/min, the resistance change remains essentially constant.

The sensor’s real-time response to a 0.3% slight strain is shown in Fig. 3d, with the tensile speed set at 300 mm/min. The enlarged view of the stretch and release processes displays that the response and recovery times are 31 and 62 ms, respectively. What’s more, as presented in Fig. 3e, the sensor’s resistance change at the same strain is almost identical, which indicates that the sensors have a high cyclic stability. It is also shown that the sensor has an extremely low detection limit, enabling it to recognize 0.1% of strains. Figure 3f is the long-term cyclicity performance of the HTF–CGF strain sensor at 50% strain and 150 mm/min rate. During the initial, intermediate, and terminal periods of the testing process, no significant signal fluctuations were observed for the six randomly selected cycles, indicating good stability and durability of the sensors. CGF is still well bonded to the HTF surface after being repeatedly stretched for hundreds of cycles (Fig. S3). Sensing units prepared using this design strategy have superior performance to other conventional design strategies (Fig. S4), paving the way for the creation of strain sensors with both low modulus and high sensitivity.

Figure S5 shows the hysteresis curves of these samples. VHB exhibits a degree of hysteresis (DH) of ~42.78% within the strain range of 0–50%, whereas HTF shows only 11.88%. This phenomenon indicates that the sensors based on the HTF can monitor the dynamic progress of VHB in real-time. It also reflects the inaccuracy of using VHB material as a sensing unit due to the large DH. Stress relaxation of the polymers remains still one of the biggest challenges facing the accuracy requirements of flexible strain sensors. No significant change is shown in the dynamic response curve at 1000 loading/unloading cycles, indicating the excellent durability of HTF (Fig. S6).

To enable the sensor to accurately and in real-time monitor the deformation of the actuator, strong adhesion between the layers is required. Therefore, plasma treatment was performed on HTF–CGF. The effectiveness of plasma treatment was demonstrated by comparing the interfacial toughness and fracture limit between VHB with TPU film, untreated HTF–CGF, plasma-treated HTF–CGF, and Polydimethylsiloxane (PDMS). The debonding resistance and toughness of four different interfaces were measured via a 90° peeling test, and the results are shown in Fig. S7. Due to the viscoelasticity of VHB, the TPU film forms surface-to-surface contact with VHB through physical adhesion, exhibiting a debonding resistance of 12.5 N/m and an interfacial toughness of 18.7 J/m^2^ during peeling. Similarly using physical adhesion, the contact between HTF–CGF and VHB is line-to-surface due to the unique form of the fibers, leading to a higher fracture resistance (22.9 N/m) and interfacial toughness (39.8 J/m^2^). The plasma-treated HTF–CGF exhibits excellent mechanical properties (debonding resistance of 73.3 N/m and interfacial toughness of 98.8 J/m^2^), outperforming other approaches. As a comparison, PDMS, a common flexible substrate, has an interfacial strength of 49.9 N/m and an interfacial toughness of 66.9 J/m^2^.

### The electroelastomer cylindrical actuator with sensing properties

To meet the application requirements for flexible and efficient movement of soft robots, flexible deformation sensing is crucial for achieving precise control of soft actuator motion. For this purpose, the HTF–CGF strain sensor is used to accurately measure the electromechanical behavior of dielectric elastomer actuators. VHB 4910 (VHB), as an electroactive polymer acrylic film with the advantages of high electromechanical coupling efficiency and high output force, is extensively used by researchers and scholars both domestically and internationally^48–50^. Therefore, the subsequent works will mainly focus on the behavior of this material in the low-strain range.

To integrate the flexible sensors onto the DEAs, the stiffness of the flexible sensor must match that of the soft actuating units to ensure that they do not affect the free deformation of the actuator. To achieve this, the mechanical properties of VHB, TPU film, TPU fibers, and HTF samples were investigated in the low strain range. Young’s modulus is defined as the slopes of tangent lines at 5% strain on the stress–strain curve^51,52^. Figure S8 shows Young’s moduli of all samples. HTF has a lower modulus of 0.155 MPa, which is lower than the modulus of VHB (0.186 MPa). The root cause of this phenomenon is that the hollow fibers further reduce the modulus of the membrane.

HTF–CGF sensing unit is attached to the DEAs to examine its influence on the electromechanical behavior of the actuator. As shown in Fig. S9, the effect of the DEAs with the sensing unit was demonstrated by comparing the actuation area strain at quasi-static and dynamic responses of two configurations: (i) DEAs without HTF–CGF, (ii) DEAs with HTF–CGF. Besides, an insulating layer must be placed between the high-voltage source and HTF–CGF to protect the sensor. Figure S9a illustrates the actuation area strain of structures #1 and #2 at different voltages. It is noteworthy that the presence of the insulating layer causes a reduction in area strain. In addition, the actuation area strain of structure #2 is smaller than that of structure #1. For example, at 6 kV, the area strain of structure #2 is ~15.2%, which is 0.88 times that of structure #1. This is because the sensor is considered a passive region relative to the actuator. Subsequently, the output characteristics of the actuator with were tested at various frequencies.

Figure S9b present the results obtained using excitation frequencies of 0.1, 1, and 10 Hz. It can be observed that structure #2 responds well to different frequencies. The actuator experiences some deformation loss under high-frequency driving, resulting in a reduction in peak values. In the continuous cycling test from 0 to 100 s, there is no obvious occurrence of mutation signals, which reveals that structure #2 has excellent cyclic stability.

**Fig. 4:**
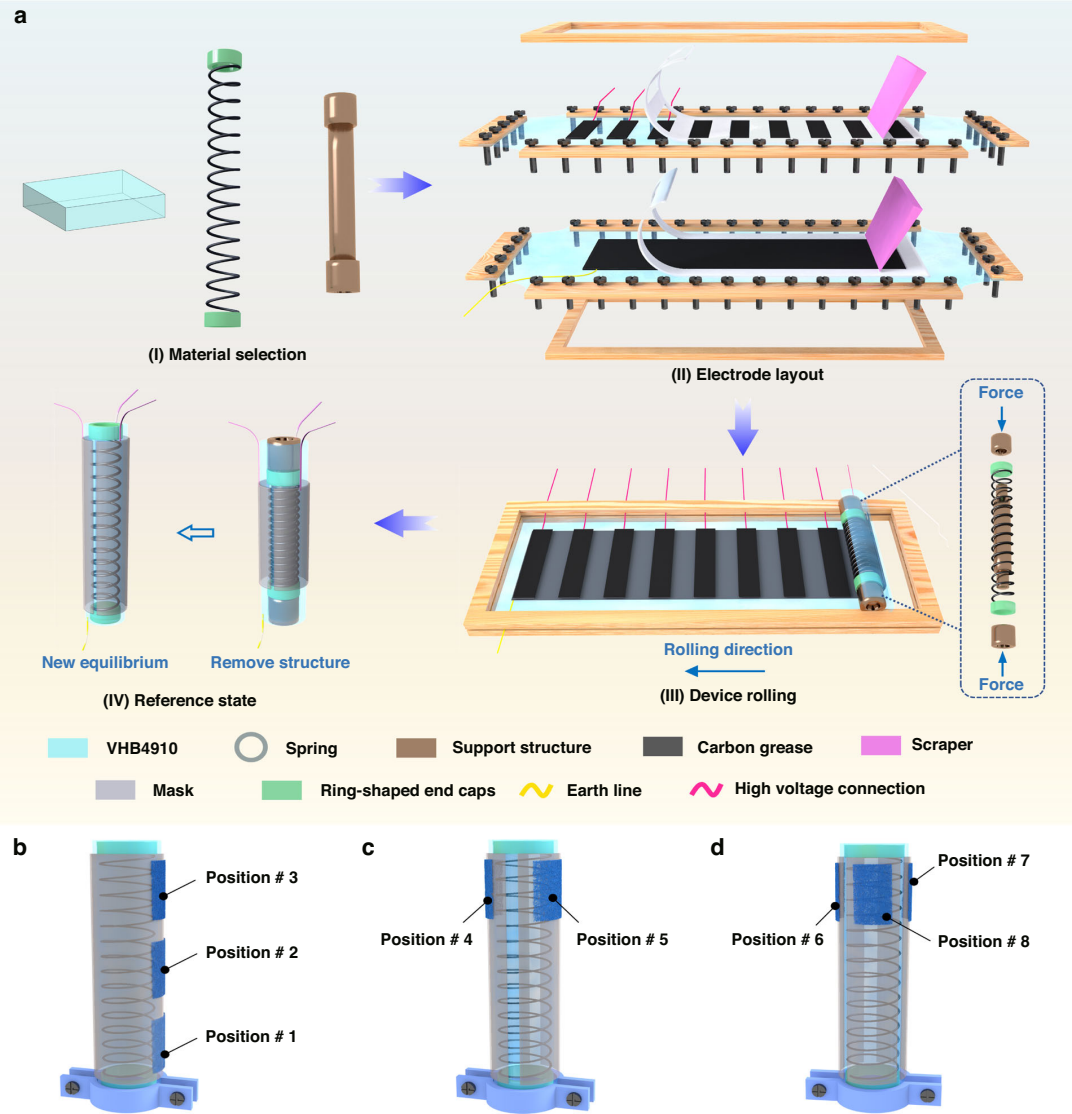
The fabrication process of SRA and its sensor installation diagram. **a** The manufacturing process of the SRA. Attachment positions of the sensor on the **b** 1DOF, **c** 2DOF, and **d** 3DOF actuators

Based on these properties, the sensor can be applied in the spring-roll actuator (SRA), the preparation of which is shown in Fig. 4a. By dividing VHB into multiple active deformation regions and reasonably arranging them around the spring, application requirement for 1, 2, or 3 degrees of freedom (1DOF, 2DOF, and 3DOF) motion can be achieved. The SRA is integrated with the HTF–CGF sensor to realize simultaneously active motion and perception function. The proper position of the sensors is crucial for accurately detecting the deformation of the actuator, and Fig. 4b–d shows the sensor positions for various actuators.

**Fig. 5:**
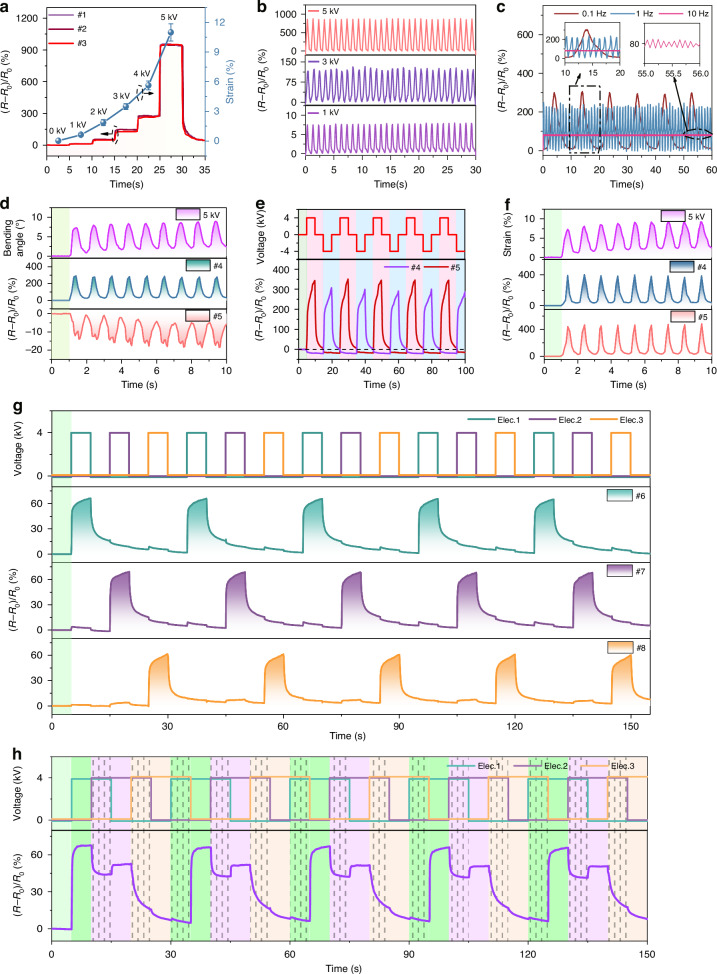
Real-time perception characteristics of the electroelastomer cylindrical actuator. **a–c**, **d–f**, and **g**, **h** are the characterizations for 1DOF, 2DOF, and 3DOF actuators, respectively. **a** Resistance changes and length strain when the actuator is subjected to a continuous voltage. Dynamic performance of the sensor under **b** actuating voltages of 1, 3, and 5 kV and **c** actuating frequencies of 0.1, 1, and 10 Hz. Sensing signals and bending angle when the actuator is bent **d** only in one direction and **e** in both left and right directions. **f** Sensing signals and length strain when both electrodes are energized simultaneously. The resistance changes when the three electrodes are energized **g** sequentially and **h** alternately

The sensor was attached to the 1DOF actuator at (i) the fixed, (ii) the middle, and (iii) the free end. Continuous voltage was applied to the 1DOF actuator, and the relative changes in sensor resistances were measured. As shown in Fig. 5a, the sensing signals at these positions remain almost identical, indicating that the 1DOF actuator exhibited homogeneous deformation. Further investigation revealed that the sensor could detect the deformation of the 1DOF actuator. Considering the ease of operation, all sensors are placed at the free end of the actuator. 1DOF actuator is subjected to cyclic test under various voltages and its sensing signal is shown in Fig. 5b. As the actuator elongation varies at different voltages, resulting in different resistance changes of the sensor. In addition, various excitation frequencies are applied to the 1DOF actuator at a constant voltage of 4 kV. As shown in Fig. 5c, at lower excitation frequencies, the charge variation on both sides of VHB becomes significant during each charging/discharging cycle, resulting in an increase in the amplitude of the actuator. The amplitude of the strain variation of the sensor was greatest at 0.1 Hz, indicating that the elongation of the actuator is greatest at this frequency. As the frequency reaches 10 Hz, the charge variation becomes very small, causing the small amplitude and high-frequency vibrations of the actuator at a fixed elongation. It was observed that the sensor still exhibits stable signal output at a high frequency of 10 Hz.

Via coupling the output signals of multiple sensors, the actuator’s direction deformation can be perceived in real-time. A 2DOF actuator capable of both bending and elongation deformations was fabricated and were attached two sensing channels to the actuator. The 2DOF actuator can generate a bending deformation as the single-sided electrode is energized. At this point, the sensor attached to the active region is stretched, while the sensor on the opposite side is compressed. Figure 5d shows the signals of the two sensing channels under loading when the actuator bends only in one direction. The resistance span shown in position #4 is much larger than that in position #5, indicating that the location of #4 is being stretched, while the location of #5 is being compressed. Applying alternating voltages to the electrode pairs on both sides of the actuator (top panel in Fig. 5e), the resistance of the two sensing channels increases in tension and decreases in compression (bottom panel in Fig. 5e). Based on this fact, when the relative resistance of one sensor increases significantly while the resistance of another sensor decreases slightly, it can be determined that the actuator is bending in the direction opposite to the one in which the resistance increases. The actuator generates elongation deformation along with both electrodes being applied voltage. At this point, both sensing channels are stretched, and their sensing signals are shown in Fig. 5f.

Attach the three sensing channels (positions #6, #7, and #8) around the 3DOF actuator, corresponding to the three electrodes (elec. 1, 2, and 3). When the three electrode pairs are energized sequentially, the signals generated by the three sensing channels are shown in Fig. 5g. Similarly, the sensor corresponding to the active region is stretched and its resistance increases, while the resistance of the other two sensors changes relatively insignificantly. For example, within one cycle, an increase in the resistance of position #6, with relatively small changes in the resistance of position #7 and position #8, indicates that the actuator was bent in the opposite direction of position #6. Subsequently, when the resistance of position #6 decreases, the change in resistance of position #7 increases while the change in resistance of position #8 is not significant, indicating that the direction of bending of the actuator shifts from the opposite direction of position #6 to the opposite direction of position #7. Under this selective electrical stimulation, the free end of the actuator generates a motion trajectory similar to a triangle. The motion direction of the actuator can also be clearly determined through the sensing signals.

To make the actuator perform more complex motions, the three electrodes of the actuator are programmed to turn on/off, causing its free end to generate a hexagonal motion trajectory. The top and bottom panels of Fig. 5h demonstrate the specific electrical stimulation program and the sensing signals of position #6, respectively. In one cycle, the sensing signal can be divided into four phases: (1) when only elec. 1 is activated, the actuator bends in the opposite direction of 6, causing the sensing signal at position #6 to increase significantly; (2) after switching to electrodes 1 and 2 working together, the actuator bends in the opposite directions of both position #6 and position #7, causing the relative resistance to decrease; (3) when elec. 2 works alone, the sensor is further stretched compared to the previous state, resulting in a relative increase in resistance; (4) subsequently, the resistance decreases until electrodes 1 and 3 work together and the sensing signal increases again. These phenomena further confirm that the sensors can accurately detect the deformation motion of the actuator.

### Potential application of a soft robot with high-precision perception

**Fig. 6:**
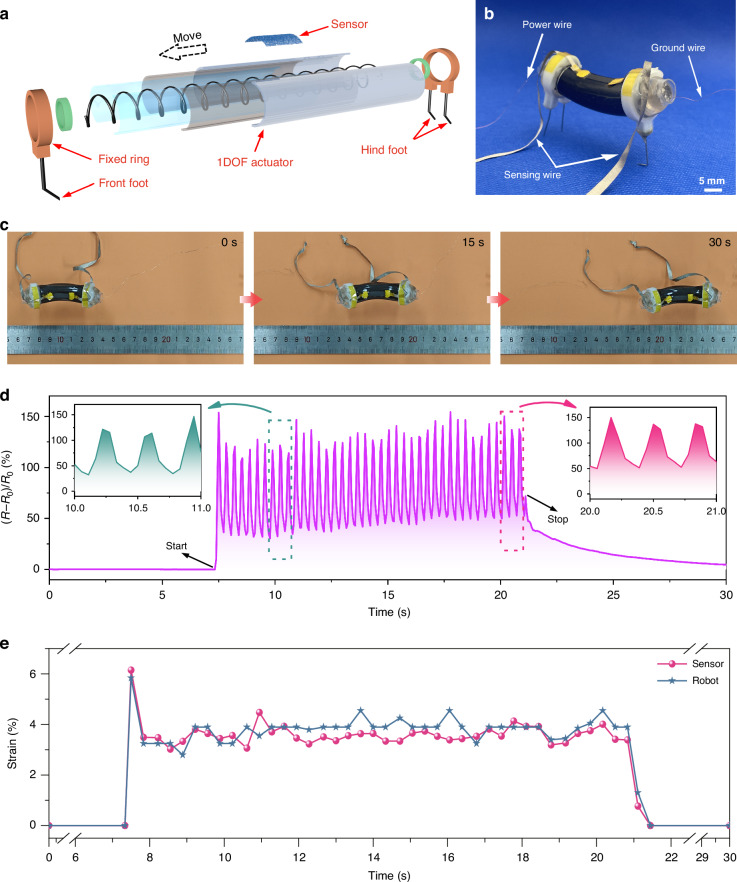
Demonstration of a soft robot with high-precision perception. **a** Basic structure. **b** Physical demonstration. **c** Real-time image of the robot moving forward. **d** Real-time sensing signals as the robot moves. **e** Real-time strain of the robot and the sensor

The inchworms move forward by adjusting the friction between the front and back feet. Inspired by this type of locomotion, we designed a soft crawling robot based on the spring-roll actuator, which is equipped with one and two anisotropic hooks on its front and back feet to guarantee its forward direction (Fig. 6a). Attaching HTF-CGF strain sensor to the robot’s abdomen endows it with sensing capabilities. Figure 6b shows a physical image of a crawling robot with high-precision perception.

As shown in Fig. 6c and Video S1, the crawling robot is subjected to a 4 kV voltage and a 3 Hz frequency with a speed of approximately 8.6 mm/s (0.13 body length per second). Figure 6d clearly shows the resistance signals of the sensing channels during the robot’s crawling process. It can be seen that there are relatively significant changes in resistance. It should be noted that due to the uneven surface roughness of the wooden board, the robot experiences variations in deformation within each alternating cycle during its crawling process. The comparison between the exact strain of the robot and the solved deformation by the sensor is shown in Fig. 6e. The variation trends of both are nearly consistent. This phenomenon further proves that the deformation of the robot can be accurately reflected in real-time by the sensor.

Define the average relative change in resistance as the average of the differences between the maximum and minimum resistance values within each alternating cycle over a period of five seconds. A constant frequency of 4 Hz was applied to the robot to investigate the crawling speed and average relative resistance change under different voltages. The robot crawling speed and the average relative resistance change are positively correlated with the voltage (Fig. S10a). This can be explained by a greater elongation for the actuators due to the increased voltage. In addition, the robot’s output properties under various frequencies were investigated in Fig. S10b. As the frequency increases, the crawling speed of the robot becomes faster, while its average relative resistance change becomes smaller. This is due to the viscoelastic properties of the actuating unit which reduces the magnitude of deformation of the actuator under high frequency excitation. These experiments demonstrate that the sensor exhibits excellent sensitivity and outstanding low detection limits in the range of 0.1–10 Hz.

## Conclusion

In summary, a low-modulus flexible microcrack-based tensile strain sensor with excellent sensing performance, good stability, and high durability has been developed. HTF was prepared through a simple coaxial electrospinning process to serve as a low-modulus (~0.155 MPa) flexible stretchable substrate. Porous graphitic flakes were produced via CO_2_ laser pyrolysis of commercial PI to act as a conductive network. Subsequently, the CGF was encapsulated on the surface of HTF using an electrostatic self-assembly process, forming a sensing unit composed of rigid graphene and flexible hollow fibers. Under increasing strain, the deformation of HTF by absorbing the mechanical energy, leads to the effective separation of interconnected graphene sheets, thereby leading to a sharp decrease in the conductive pathways. This synergistic strategy facilitates a good disconnection/reconnection mechanism between the graphene sheets, thereby improving the sensing performance. Consequently, the HTF–CGF strain sensor exhibits a gauge factor up to 220.3 (25% < *ε* < 50%), a rapid response/recovery behavior (31/62 ms), and a low detection limit (0.1%). The sensors were applied to DEAs to monitor their deformation. Profiting from the hollow fiber configuration, the mechanical properties between HTF and the actuation unit are more closely matched. Besides, the difference in area strain between the actuator with and without sensors is only 12%. Furthermore, applying the sensor to the spring-roller actuators allows for precise sensing of their deformation modes and directions. Finally, a crawling robot based on the spring-roller actuator was developed, and the deformation of the robot has been successfully detected in real-time. This research will not only advance the development of intelligent soft robots but also provide more perspectives in human–robot interaction, medical engineering, soft bionics, and other related fields.

## Material and methods

### Preparation of HTF by drum electrospinning

Firstly, TPU pellets (Bayer MaterialScience) were dissolved in a mixed solution of DMF (Shanghai Macklin Biochemical Co., Ltd) and acetone (with a mass ratio of 1:1) and stirred thoroughly to obtain a 30 wt% TPU solution as the shell solution. Commercial glycerol (Shanghai Macklin Biochemical Co., Ltd) was used as the core solution for coaxial electrospinning. Then, TPU solution and glycerin were injected from the outer and inner channels of the coaxial nozzle at an injection rate of 500 and 60 μL/h, respectively. The syringe was placed at a distance of 15 cm from the collecter. The voltage was set to 12 kV and the collection time was 4 h. The membrane, after electrostatic spinning was immersed in deionized water for 8 h. Finally, the hollow fibers were obtained.

### Preparation of CGF

A CO_2_ infrared laser machine (JHCV-30, Wuhan Jinhuo Laser Technology Co., Ltd.) was used to sinter the PI film with a 100 μm thickness. The base laser processing parameters were as follows: laser speed (85 mm/s), laser frequency (10 kHz), and power rate (25%). Then, the laser scanning patterns were drawn using computer-aided software. Gently peel off the laser-scanned product using a knife.

### Preparation of HTF-CGF strain sensor

Firstly, the CTAB (Shanghai Macklin Biochemical Co., Ltd) solution was prepared by dissolving CTAB particles (25 mg) into deionized water (25 ml) and ultrasonicating for 1 h. Then, the SDS/CGF solution was also prepared by dissolving SDS particles (25 mg) and CGF (25 mg) into the deionized water (25 ml) and ultrasonicating for 1 h. Placed HTF obtained from coaxial electrospinning into the CTAB solution and ultrasonicating for 30 min. Then, it transferred HTF to the SDS/CGF solution and stirred for 15 min. After that, the films were washed with deionized water and then dried naturally. HTF-CGF sensing unit was obtained. Finally, the wires are adhered to both sides of the HTF–CGF sensing unit with silver paste and cured naturally. HTF-CGF strain sensor was completely fabricated.

### Rigorous treatments of HTF-CGF sensing unit

The sensing units were treated in two different environments, including (1) magnetic stirring in a saline solution at 60 °C and 500 rpm or (2) ultrasonic washing in deionized water.

### Preparation of the electroelastomer cylindrical actuator

The electroelastomer cylindrical actuator is composed of a spring, a dielectric elastomer film coiled around the spring, ring-shaped end caps at the ends of the spring, and carbon grease coated on the film as the flexible electrodes. The spring is pre-compressed via the support structure consisting of the nuts, the screw, and the ring-shaped end caps. First, two biaxially pre-stretched VHB (3M Company) films were laminated to form a bi-layer membrane that has an electrode layer at the top, middle, and bottom respectively. However, one electrode layer was prepared due to the contact between the top and bottom electrodes during the rolling process. In this work, the top electrode layer was fabricated. Additionally, the middle electrode was coated as a continuous rectangle to improve manufacturing efficiency. The top electrode was composed of multiple uniformly distributed small rectangular electrodes (1DOF, 2DOF, and 3DOF actuators have 1, 2*N*, and 3*N* rectangular electrodes, respectively, where *N* is the number of wrapping turns.). And an inactive gap of 2 mm must be maintained between these electrodes to ensure electrical insulation. Different patterned electrodes were scraped onto the pre-stretched film. Then, the film of the appropriate size was uniformly wrapped around the compressed spring and support structure. Finally, the entire preparation process of the actuator was completed after removing the support structure and using the ring-shaped end caps inside the actuator as spring ends.

### Preparation of the soft robot

Fixed rings were printed by the PμSL 3D printer (S240, Boston Micro Fabrication Material Technology Inc., Shenzhen, China) for connecting pins and actuators. Placed the fixed rings with one and two pins on the front and back feet of the robot, respectively. Install the two fixed rings at each end of the actuator. Then, the soft robot is successfully prepared.

### Characterization

A scanning electron microscope (JSM-IT500A, Japan) was used to characterize HTF-CGF. The surface chemical compositions and structures were further characterized by a high-resolution X-ray Photo-electron Spectroscopy (XPS, Escalab 250 Xi+, Thermo Fisher Scientific), Raman spectrometer (lDSPeC ARCTlC), high-resolution X-ray Diffractometer (XRD, XRD-7000, Shimadzu), and Fourier Transform Infrared Spectroscopy spectrometer (FT-IR, Nicolet iS10, Thermo Nicolet). Impedance performance was conducted using the electrochemical workstation (PGSTAT204, Autolab). Dynamic mechanical analysis was executed on a rotational rheometer (DHR-2). All samples were tested for mechanical properties using the universal testing machine (E43.104, China).

## Supplementary information

Supporting Video

Supporting information
