# Supplementary material for: Hollow fiber-based strain sensors with desirable modulus and sensitivity at effective deformation for dexterous electroelastomer cylindrical actuator
Source: Microsyst Nanoeng. 2025 Feb 27;11:34. doi: 10.1038/s41378-025-00878-7 (PMC11865588; doi:10.1038/s41378-025-00878-7)
Supplement: Supplementary file 2 — Supporting information [file 41378_2025_878_MOESM2_ESM.doc]

**Supporting information**

**Hollow fiber-based strain sensors with desirable modulus and sensitivity at effective deformation for dexterous electroelastomer cylindrical actuator**

Yang Zhang^1^, Keqi Deng^1^, Tingting Shen^1^, Yong Huang^1^, Zhenjin Xu^1^, Jinhui Zhang^2^, Hang Jin^1^, Xin Liu^1^, Lida Xu^1^, Lianjie Lu^1^, Shiying Li^3^, Daoheng Sun^1^, Dezhi Wu^1, *^

^1^ Pen-Tung Sah Institute of Micro-Nano Science and Technology, Xiamen University, Xiamen 361005, China

^2^ Department of Mechanical & Electrical Engineering, Xiamen University, Xiamen 361005, China

^3^ Department of Ophthalmology, the First Affiliated Hospital of Xiamen University, School of Medicine, Xiamen, Fujian Province,361005, China

^*^ Corresponding author

E-mail address: [wdz@xmu.edu.cn](mailto:wdz@xmu.edu.cn)


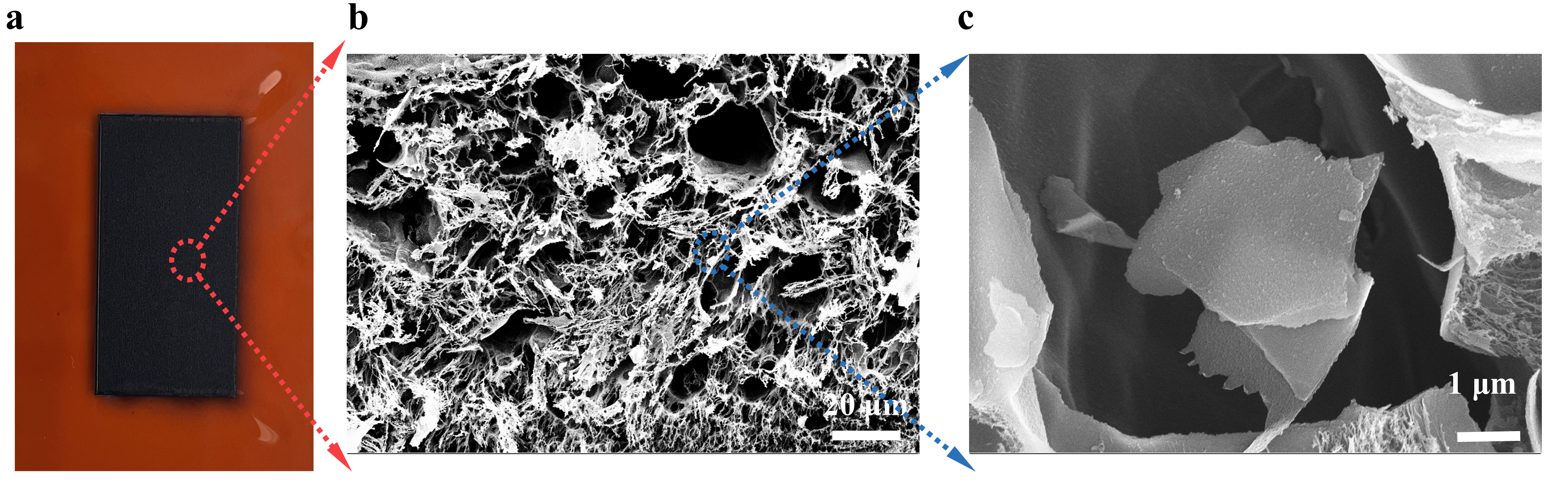


**Fig. S1.** **a** Photograph of CGF. SEM image of **b** top-view and **c** enlarged view of CGF


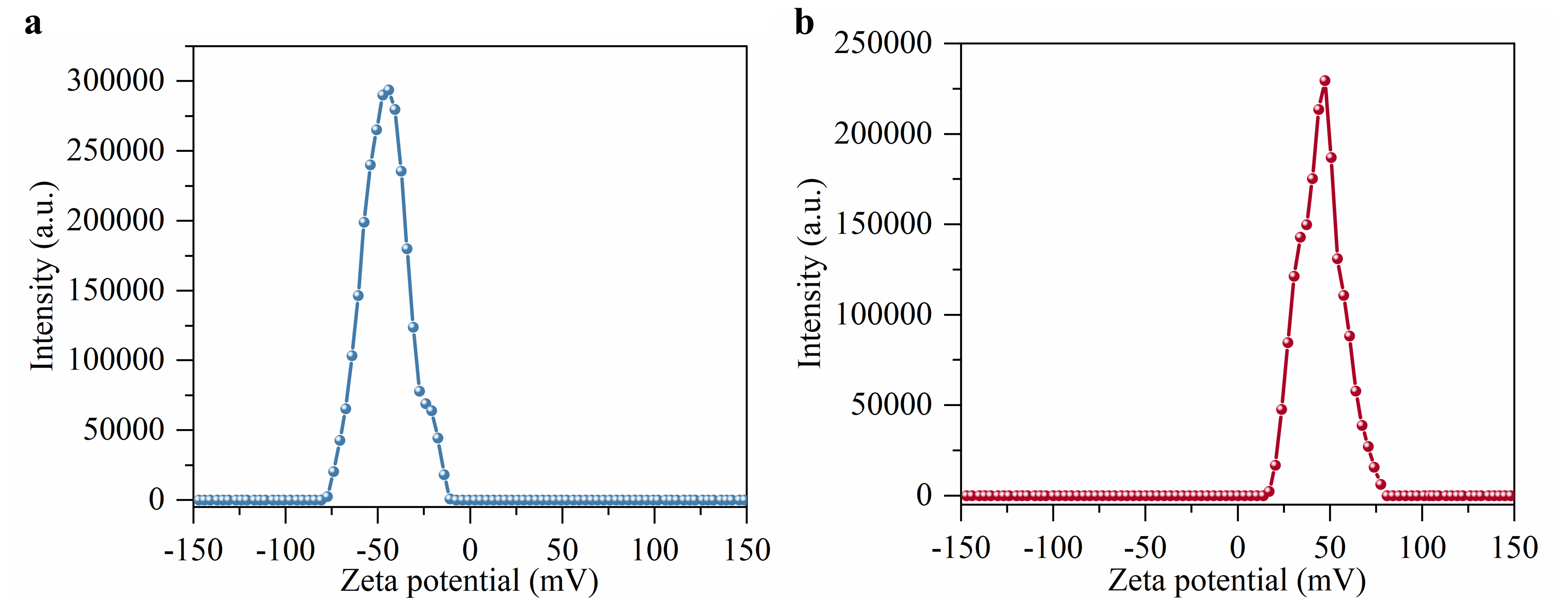


**Fig. S2.** The zeta potential of **a** SDS/CGF solution and **b** CTAB solution


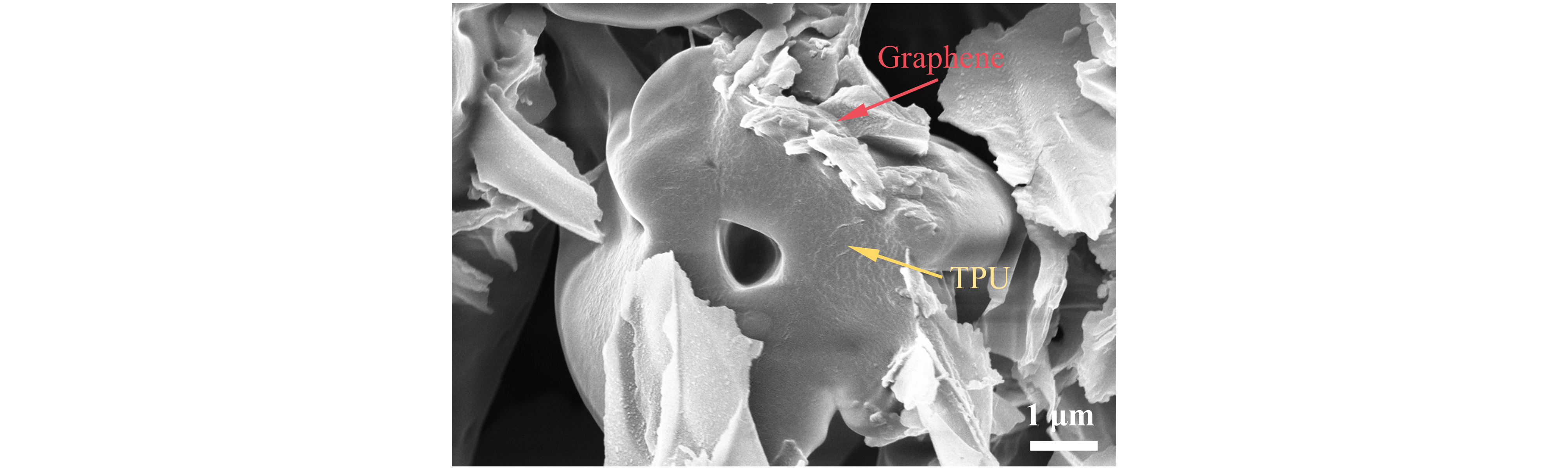


**Fig. S3.** The cross-sectional SEM images of the HTF-CGF sensing unit after being repeatedly stretched for hundreds of cycles


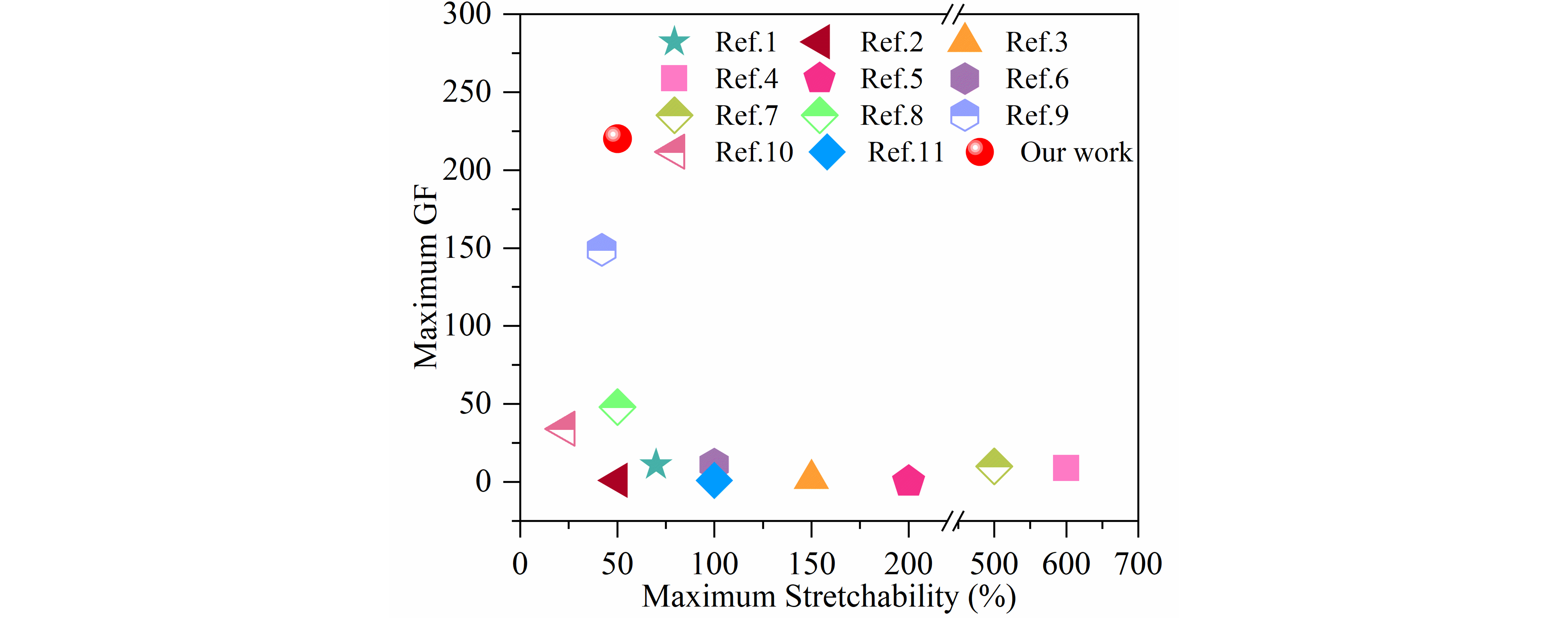


**Fig. S4.** Comparison of the maximum GF and stretchability of strain sensors recently reported in the literature^1-11^ with our sensor


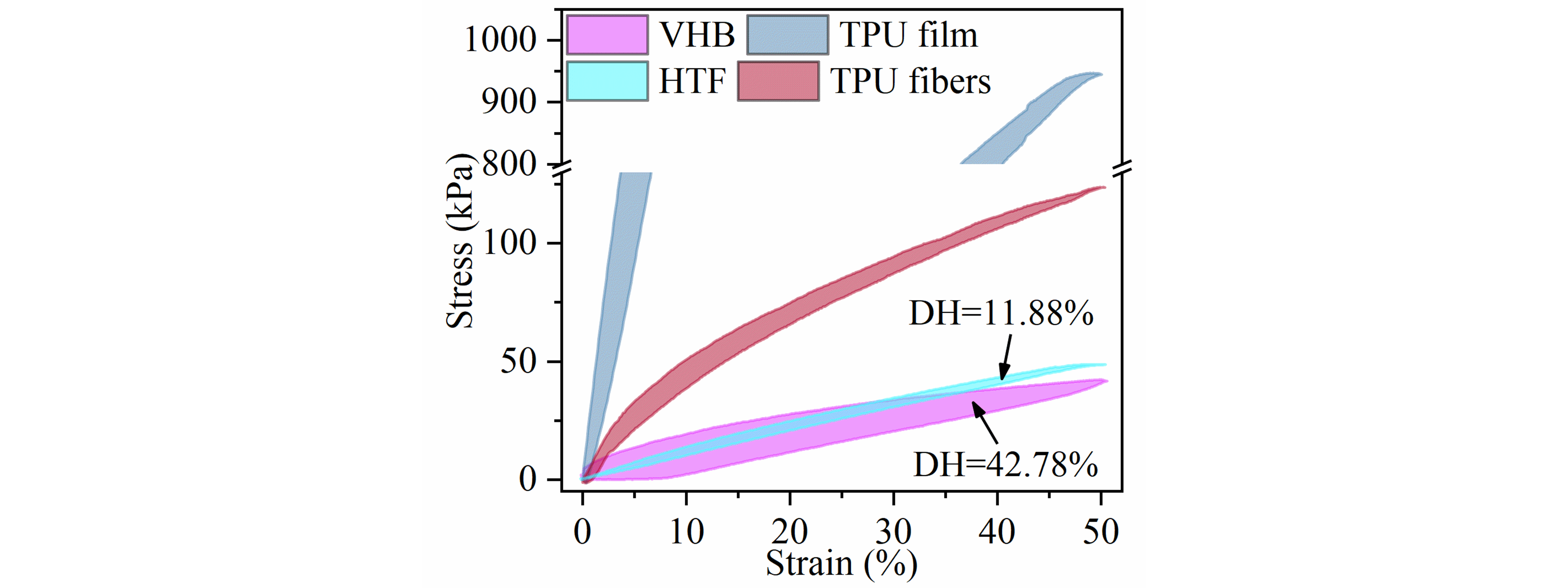


**Fig. S5.** The cyclic stress–strain curves of VHB, TPU film, TPU fibers and HTF at a stretch rate of 100 mm/min. The area encircled by the curve indicates the dissipated mechanical energy


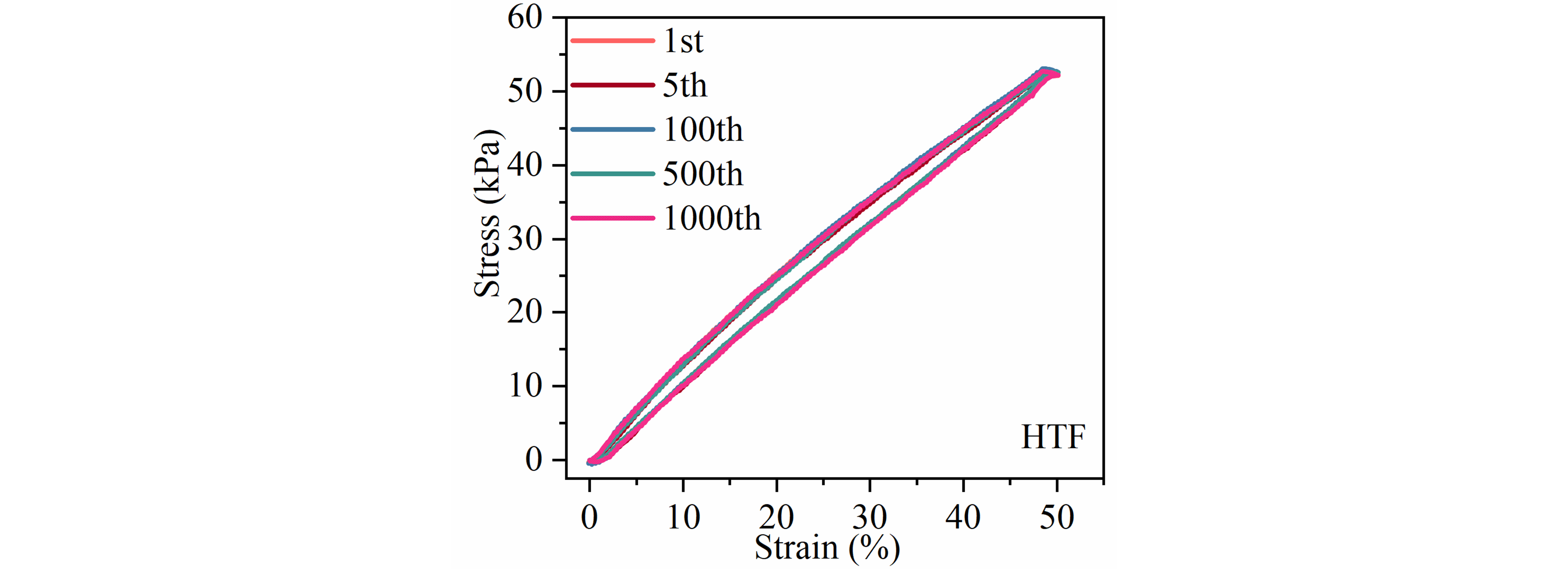


**Fig. S6.** Stress-strain curves of HTF during the endurance test at 50 % strain for 1000 cycles


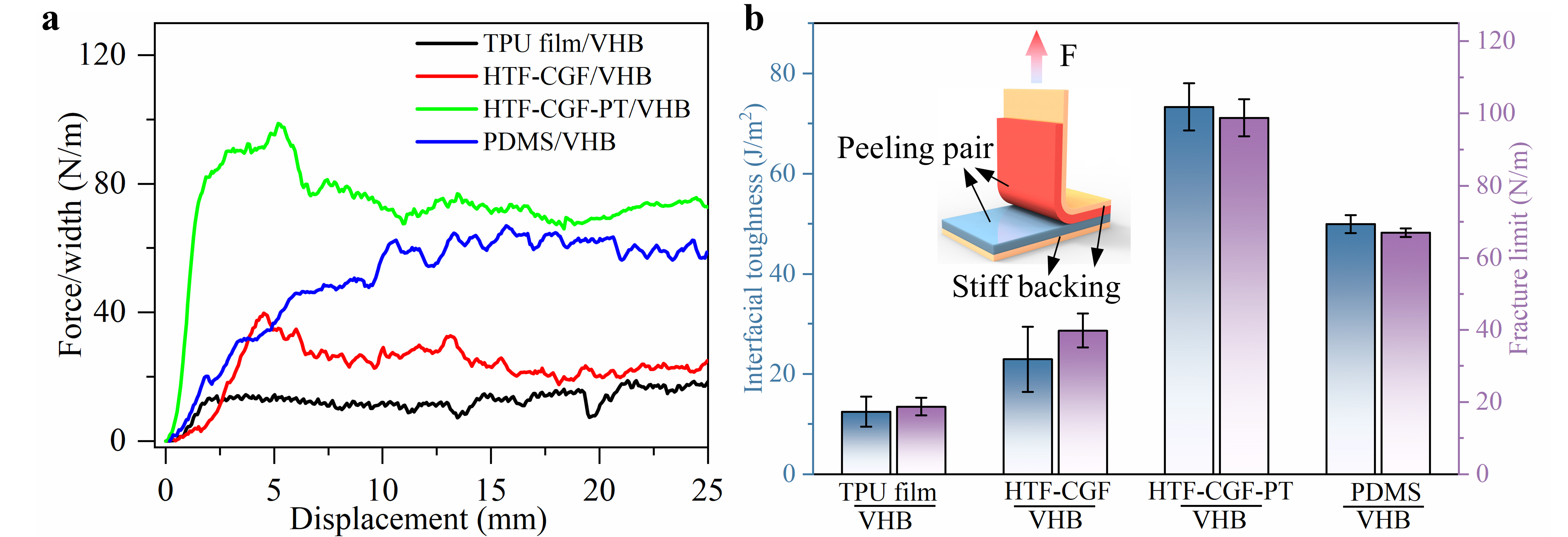


**Fig. S7.** **a** Peel force as a function of displacement for the four different interfaces, and **b** interfacial toughness and fracture limit of the four different interfaces


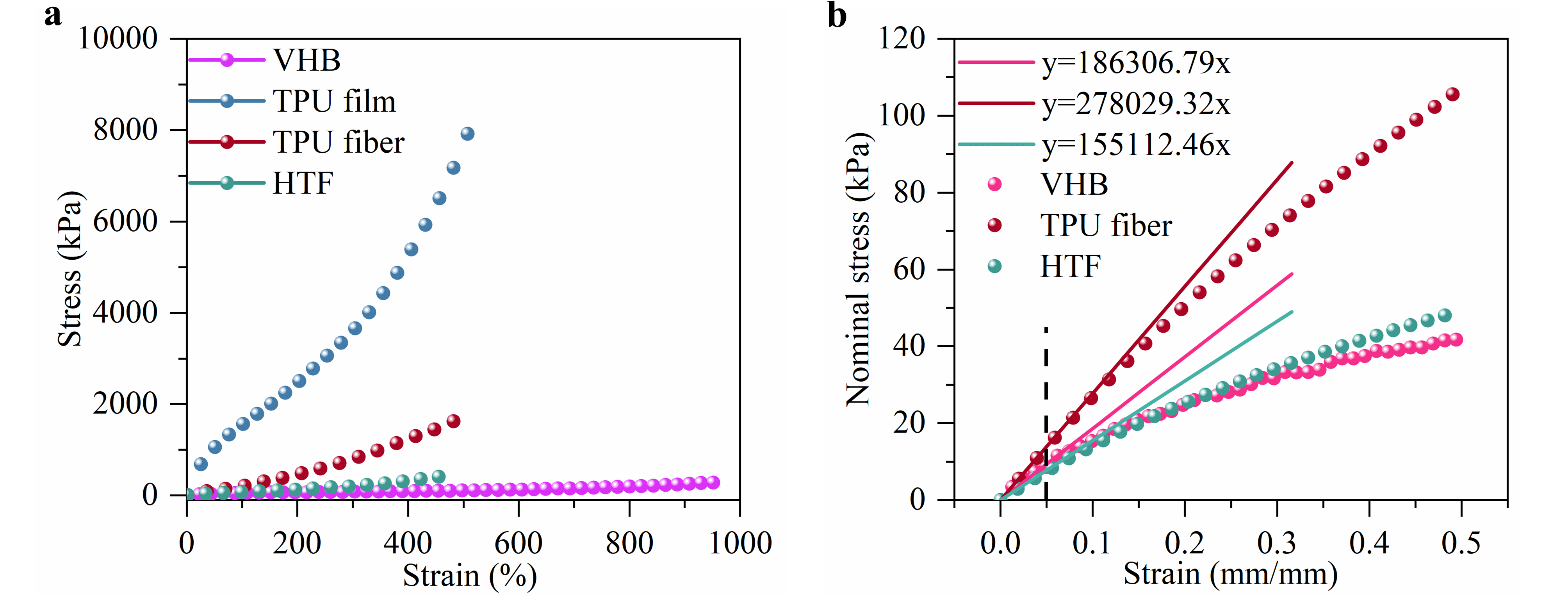


**Fig. S8.** **a** Nominal stress versus strain curves, and **b** Young’s moduli of VHB 4910, TPU film, TPU fibers and HTF at a stretch rate of 100 mm/min


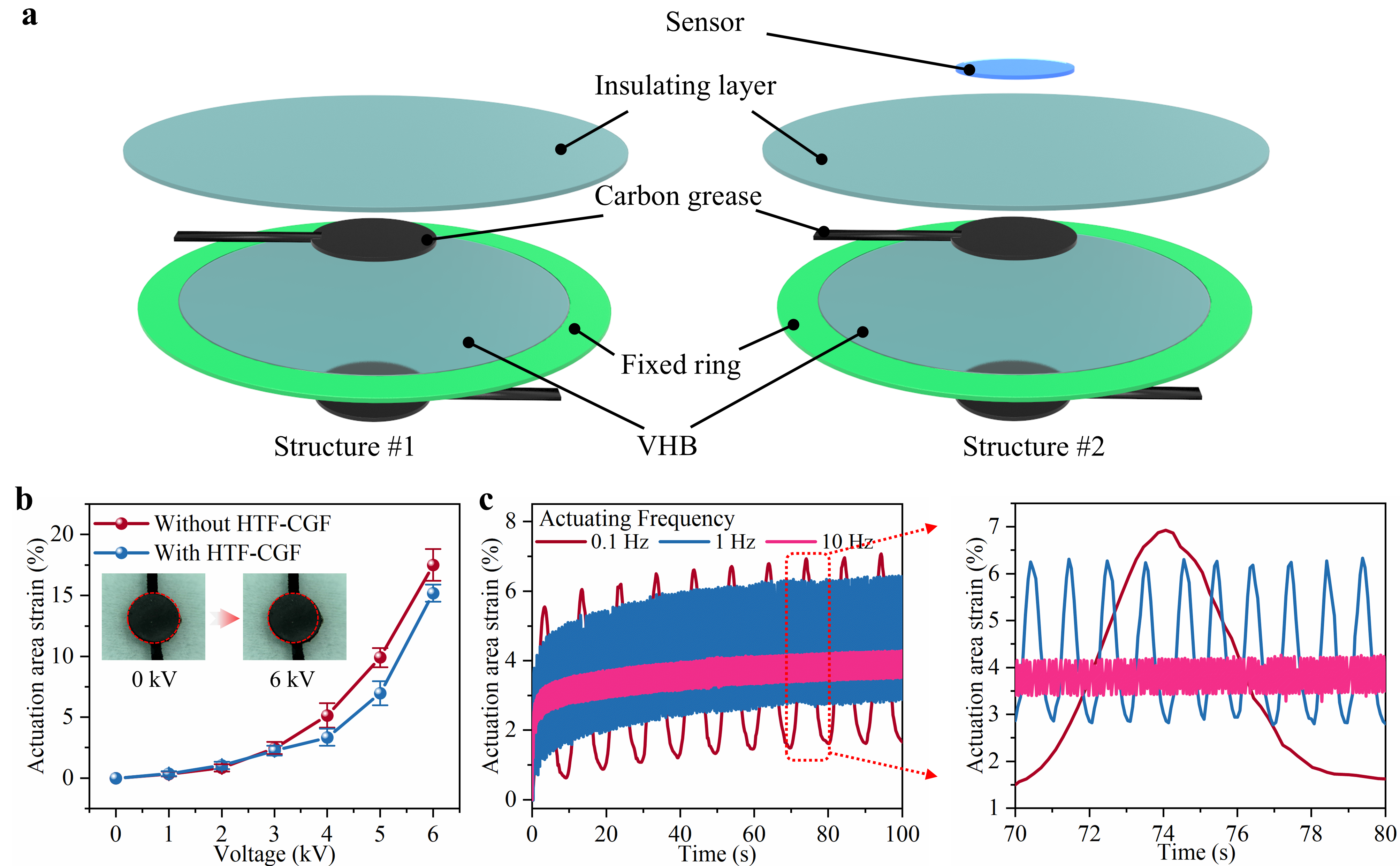


**Fig. S9**. **a** Schematic of two device configurations. **b** Dependence of actuation area strain on the voltage for VHB with/without HTF-CGF. The inset shows the schematic diagrams illustrating the electro-actuation deformation process for VHB with HTF-CGF samples. **c** Frequency response of VHB with HTF-CGF to large drive signals (4 kV) in the range of 0.1, 1 and 10 Hz


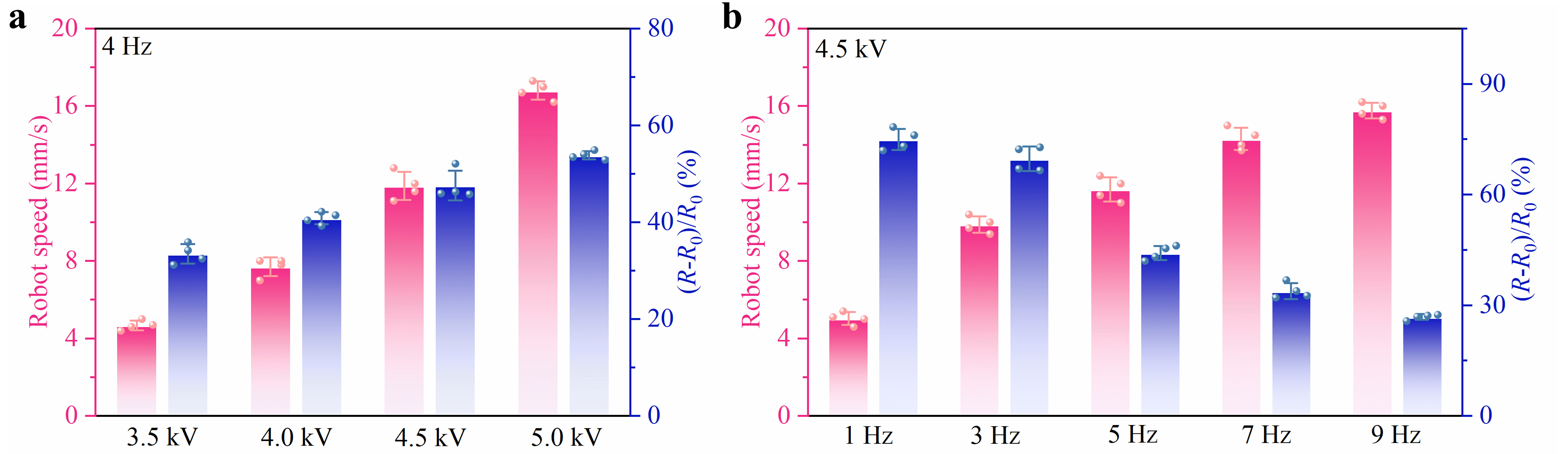


**Fig. S10**. Robot speed and sensing signals at **a** various voltages and **b** various frequencies

**Reference:**

1. Ma, S. et al. Responsive soft actuators with MXene nanomaterials. **2**: e20230026 (2024).

2. Yang, Y. et al. High-throughput printing of customized structural-color graphics with circularly polarized reflection and mechanochromic response. **7**: 2091-107 (2024).

3. Ma, J. et al. Mechanochromic and ionic conductive cholesteric liquid crystal elastomers for biomechanical monitoring and human–machine interaction. **11**: 217-26 (2024).

4. Li, X. et al. Mechanochromic and Conductive Chiral Nematic Nanostructured Film for Bioinspired Ionic Skins. **17**: 12829-41 (2023).

5. Ma, S. et al. Highly Stretchable and Conductive MXene-Encapsulated Liquid Metal Hydrogels for Bioinspired Self-Sensing Soft Actuators. **34**: 2309899 (2024).

6. Xue, P. et al. Highly Conductive MXene/PEDOT:PSS-Integrated Poly(N-Isopropylacrylamide) Hydrogels for Bioinspired Somatosensory Soft Actuators. **33**: 2214867 (2023).

7. Luo, G. et al. Highly Stretchable, Knittable, Wearable Fiberform Hydrovoltaic Generators Driven by Water Transpiration for Portable Self-Power Supply and Self-Powered Strain Sensor. **20**: 2306318 (2024).

8. Luo, G. et al. Highly conductive, stretchable, durable, breathable electrodes based on electrospun polyurethane mats superficially decorated with carbon nanotubes for multifunctional wearable electronics. **451**: 138549 (2023).

9. Yang, H. et al. Computational design of ultra-robust strain sensors for soft robot perception and autonomy. **15**: 1636 (2024).

10. Shi, X. et al. Interfacial Click Chemistry Enabled Strong Adhesion toward Ultra-Durable Crack-Based Flexible Strain Sensors. **33**: 2301036 (2023).

11. Guo, Y. et al. Multifunctional Hydrogel Sensor with Curved Macro Cracks: A Strategy for High Sensitivity and Wide Detection Range. **33**: 2306820 (2023).
